# Characterization of exogenous αSN response genes and their relation to Parkinson’s disease using network analyses

**DOI:** 10.3389/fphar.2022.966760

**Published:** 2022-09-30

**Authors:** Zahra Nayeri, Farhang Aliakbari, Farzaneh Afzali, Soha Parsafar, Ehsan Gharib, Daniel E. Otzen, Dina Morshedi

**Affiliations:** ^1^ Department of Bioprocess Engineering, Institute of Industrial and Environmental Biotechnology, National Institute of Genetic Engineering and Biotechnology, Tehran, Iran; ^2^ Department of Molecular Medicine, Institute of Medical Biotechnology, National Institute of Genetic Engineering and Biotechnology, Tehran, Iran; ^3^ Molecular Medicine Research Group, Robarts Research Institute, Schulich School of Medicine and Dentistry, University of Western Ontario, London, ON, Canada; ^4^ Department of Biomedical and Molecular Sciences, Queen’s University, Kingston, ON, Canada; ^5^ Department of Chemistry and Biochemistry, University de Moncton, Moncton, ON, Canada; ^6^ Interdisciplinary Nanoscience Centre (iNANO) and Department of Molecular Biology and Genetics, Aarhus University, Aarhus, Denmark

**Keywords:** α-synuclein, irinotecan, entrectinib, COVID-19, Parkinsion’s disease, systems biology

## Abstract

Despite extensive research, the molecular mechanisms underlying the toxicity of αSN in Parkinson’s disease (PD) pathology are still poorly understood. To address this, we used a microarray dataset to identify genes that are induced and differentially expressed after exposure to toxic αSN aggregates, which we call *exogenous αSN response* (EASR) genes. Using systems biology approaches, we then determined, at multiple levels of analysis, how these EASR genes could be related to PD pathology. A key result was the identification of functional connections between EASR genes and previously identified PD-related genes by employing the proteins’ interactions networks and 9 brain region-specific co-expression networks. In each brain region, co-expression modules of EASR genes were enriched for gene sets whose expression are altered by SARS-CoV-2 infection, leading to the hypothesis that EASR co-expression genes may explain the observed links between COVID-19 and PD. An examination of the expression pattern of EASR genes in different non-neurological healthy brain regions revealed that regions with lower mean expression of the upregulated EASR genes, such as substantia nigra, are more vulnerable to αSN aggregates and lose their neurological functions during PD progression. Gene Set Enrichment Analysis of healthy and PD samples from substantia nigra revealed that a specific co-expression network, “TNF-α signaling via NF-κB”, is an upregulated pathway associated with the PD phenotype. Inhibitors of the “TNF-α signaling via NF-κB” pathway may, therefore, decrease the activity level of this pathway and thereby provide therapeutic benefits for PD patients. We virtually screened FDA-approved drugs against these upregulated genes (*NR4A1*, *DUSP1*, and *FOS*) using docking-based drug discovery and identified several promising drugs. Altogether, our study provides a better understanding of αSN toxicity mechanisms in PD and identifies potential therapeutic targets and small molecules for treatment of PD.

## Highlights

1) Identifying mechanisms and critical pathways underlying αSN toxicity could lead to the development of effective treatments of PD.

2) Exogenous αSN toxic aggregates lead to alteration in the expression of a group of genes that we called exogenous αSN response (EASR) genes, which have functional associations with key genes involved in PD.

3) The upregulated and downregulated EASR genes display altered expression levels across 9 brain regions that are associated with vulnerability to αSN aggregation in PD.

4) Entrectinib and Irinotecan are FDA-approved drugs that bind and inhibit the *NR4A1*, *DUSP1*, and *FOS* proteins, and therefore potentially decrease the activity of αSN toxicity-related pathways in the substantia nigra.

## Introduction

Parkinson’s disease (PD) is the second most destructive neurodegenerative disorder. Its prevalence has grown dramatically worldwide (Dorsey et al., 2018). The most well-known symptoms of PD are depleted dopaminergic neurons in substantia nigra, reduction of dopamine levels in the striatum, and production of intracellular proteinaceous amyloid aggregates, called Lewy bodies (LBs) and Lewy neurites (LNs), which consist primarily of α-synuclein (αSN). αSN is a conserved presynaptic protein with a molecular weight of 140 kDa and is very abundant (with a number density of more than 1% of total proteins in the brain (Breydo, Wu and Uversky, 2012). αSN is an intrinsically disordered protein that can adopt multiple structures under various physiological conditions, including self-assembling into toxic amyloid aggregates. Amyloid aggregation of αSN is a pivotal process in the progression of PD and other related disorders. Intercellularly aggregated αSN can spread from cell to cell via an exosome-based delivery system, microtubule-based transport, or receptor-mediated endocytosis (Christensen et al., 2016; Emmanouilidou and Vekrellis, 2016; Dieriks et al., 2017). Penetration of extracellularly-aggregated αSN into neurons induces αSN fibrillation in the infected neurons and, consequently, spreads neurodegeneration around the brain (Hornedo-Ortega et al., 2016).

It has been proposed that intercellular transmission of αSN to neighboring cells and across brain regions follows a spatiotemporal pattern, which begins from the brainstem and later spreads to more rostral sites of the brain (Braak et al., 2003). Although the spread of αSN explains PD-related pathology and symptom progression to an extent, the reason why certain kinds of neurons and particular parts of the brain are distinctly further vulnerable to synucleinopathy is not yet fully understood (Henderson et al., 2019).

Furthermore, the spatiotemporal pattern of αSN spread correlates with changes in gene expression in particular regions of the brain (Nowakowski et al., 2017; Keo et al., 2020). According to gene expression pattern analyses, these spatial expression patterns could potentially be involved in PD pathophysiology (Guo et al., 2019). Also, some PD-related genes have brain region-specific expression patterns (Keo et al., 2020).

Characterizing the distinct expression patterns in different parts of the brain helps to identify the factors contributing to cell vulnerability to αSN aggregates. Here, we identified what we call *exogenous αSN response* (EASR) genes using a microarray dataset collected using SH-SY5Y cells that were treated with αSN aggregates. We then applied system biology approaches to find associations of EASR genes with PD-related genes.

To this end, we developed and analyzed two different network models. First, we obtained PD-related genes and concentrated on the protein-protein interaction network (PPI) between these genes and the EASR genes to study the functional connections between PD and αSN response at the protein level. Then, we collected expression data of healthy brains from different data sources and, at the expression level, studied their transcriptomes and compared the expression patterns of EASR genes between the distinct brain regions. Subsequently, we developed the second network model by constructing the co-expression network of EASR genes for each studied brain region and examined pathways that were enriched in these networks. We also studied the co-expression network of two brain regions, the substantia nigra and amygdala, between PD and control phenotypes, and characterized the enhanced activity of the “TNF-α signaling via the NF-κB” pathway as a significant pathway in substantia nigra-mediated αSN neurotoxicity during PD. Identifying this critical pathway could lead to the development of effective treatment for PD patients. Furthermore, our studies yielded important insights into the molecular mechanisms underlying αSN toxicity and PD progression. Finally, we applied docking-based virtual screening to identify approved drugs that could potentially be repurposed for PD treatment.

## Methods

### Data collection

**FIGURE 1:**
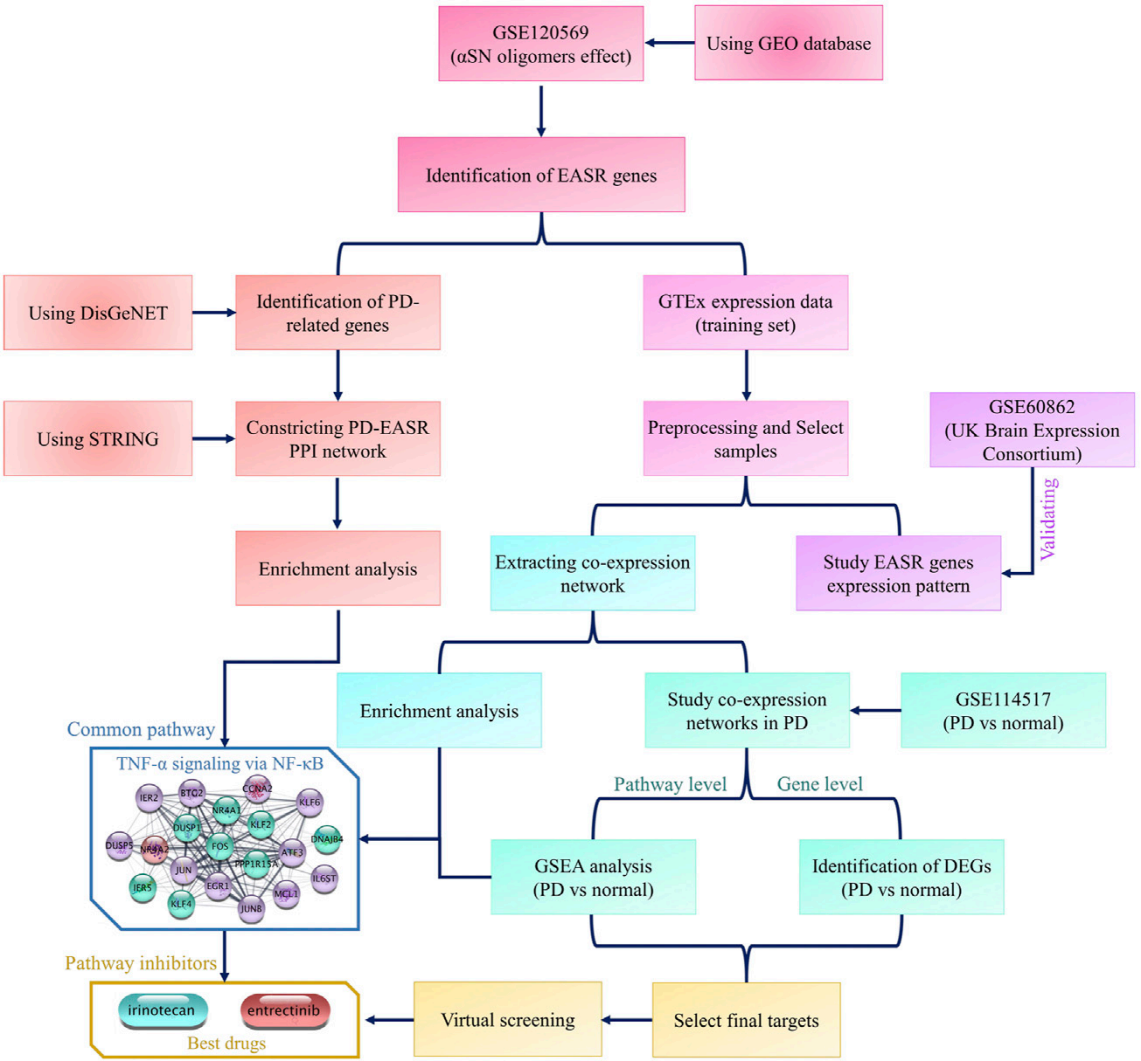
Flowchart of this study. The flowchart represents all datasets and analyses that were used in the study.

Multiple datasets have been used in this study. All datasets and analyses are summarized in the flowchart in Figure 1. To identify the exogenous αSN response genes (EASR genes), we used the GSE120569 dataset to detect transcriptional changes induced by exogenous αSN. This dataset released expression data of SH-SY5Y cells, which were exposed to the special aggregate of αSN that produced during fibrillation condition (Lin et al., 2018). Differentially expressed genes (DEGs) with a |fold change| > 1.5 and *p*-value < 0.05 were considered to be part of the EASR gene set.

To investigate the patterns of EASR gene expression in different parts of the brain, we used expression data from the Genotype-Tissue Expression (GTEx) project that collected samples from 54 non-diseased tissue sites across nearly 1,000 individuals. After preprocessing, we selected samples that related to 9 different brain regions, including the amygdala (by 68 samples), anterior cingulate cortex (by 83 samples), caudate (by 109 samples), cerebellum (by 118 samples), frontal cortex (by 95 samples), hippocampus (by 84 samples), hypothalamus (by 82 samples), putamen (by 79 samples), and substantia nigra (by 57 samples). The UCSC Xena Browser (https://xenabrowser.net/datapages/) was used to download the GTEx expression data.

The UK Brain Expression Consortium (UKBEC) (http://www.braineac.org) datasets include microarray expression data of healthy (non-neurological) brain samples, which is available through accession code. The UKBEC data cover 4 of the 9 brain regions of interest in our study, including the frontal cortex, hippocampus, putamen, and substantia nigra. These datasets were used as validation sets.

Subsequently, we used the GSE114517 dataset to study the expression patterns of EASR in different parts of the brain regions obtained from PD patients. This dataset contained samples that match with two GTEx brain regions, substantia nigra and amygdala.

Eventually, we also used PanglaoDB (https://www.panglaodb.se/index.html) to obtain cell type gene expression markers.

### Network construction and topological analysis

All network illustrations and analyses were performed using Cytoscape version 3.7.2 (Shannon et al., 2003). The methodology used was previously reported (Parsafar et al., 2020). To identify the PD-related genes, we used the DisGeNET platform (version 7.0, https://www.disgenet.org/home/), which contains 1,134,942 gene-disease associations (Piñero et al., 2015). Subsequently, a protein-protein interaction (PPI) network between EASR and PD-related genes was constructed using the stringApp plugin. A confidence score ≥0.4 and a maximum number of interactors = 0 were used as cutoffs (Doncheva et al., 2018), so that no additional protein be added to the networks. The topological characteristics of the resulting networks such as degree, betweenness, or average shortest path length, were examined using the NetworkAnalyzer and cytoHubba plugins (Assenov et al., 2008; Chin et al., 2014). Finally, the EASR co-expression network for each brain region was constructed using Inetmodels (https://inetmodels.com/). The node limit and the edge pruning parameter (-Log10 Adjusted *p*-value) were taken as 25 and 2, respectively.

### Gene expression analysis

To process PD RNA-seq data, we applied the commonly used RNA-seq pipeline (Trimmomatic, HISAT2, HTSeq, and DESeq2). First, we used Trimmomatic to obtain clean data (clean reads) by trimming the paired-end raw reads and removing the read containing adapter, poly-N, and low-quality bases (Bolger, Lohse and Usadel, 2014).

Then, the cleaned data were mapped to the indexed reference genome by applying Hisat2. HTSeq was then applied to count the reads mapped to each gene (Anders, Pyl and Huber, 2015). Moreover, sample-level quality control was performed using Principal Component Analysis (PCA) to identify any sample that needed to be excluded prior to performing the expression analysis. Finally, we performed differential gene expression analysis using the DESeq2 package in R version 4.0.3 (Love, Huber and Anders, 2014). Genes with *p*-value < 0.05 and |fold change| > 1.5 were considered as a final DEGs.

### Gene functional annotation

To further understand the biological functions of the proteins involved in each network, we ran Hallmark pathway and Gene Ontology (GO) analyses, including Molecular Function (MF), Biological Process (BP), and Cellular Component (CC) enrichment analyses using the enrichR and clusterProfiler R packages (Yu et al., 2012). We used a threshold *p*-value < 0.05 to identify statistically significant pathways and GO functions. To examine pathway enrichment between control and PD conditions, Gene Set Enrichment Analysis (GSEA) was performed using the Broad Institute’s Molecular Signatures Database (MSigDB Hallmark) and GSEA software (v4.1.0) (Subramanian et al., 2005; Liberzon et al., 2011, 2015). In addition, in this study, a false discovery rate (FDR) < 0.05 was used to determine statistically significant enrichments. Results of these analyses were plotted in R 3.6.1 using ggplot2 (Wickham, 2011) and using the Circular R package (Lund et al., 2017).

### Statistical analyses

All statistical analyses were performed with R version 4.0.3 and the Bioconductor http://bioconductor.org/ packages. Gene-gene expression-based correlation was performed using the https://CRAN.R-project.org/package=Hmisc/ Hmisc and Corrplot R packages https://CRAN.R-project.org/package=corrplot/ to calculate Spearman’s correlation and to perform visualization, respectively. We applied Corrplot with hierarchical clustering and set the significance level (*p*-value) to 0.01. In addition, we used the Complexheatmap package https://jokergoo.github.io/ComplexHeatmap-reference/ to visualize all heatmaps. We also employed ggplot2 to apply analysis of variance (ANOVA) and Student’s t-test to assess and visualize the statistical difference in multi and two groups, respectively. A *p*-value < 0.05 was considered a significant level.

### Virtual screening of FDA approved drugs

We performed docking-based virtual screening to find new inhibitors that could target the pathways and proteins that were identified in our analyses. To this end, molecular structures of drugs that were approved by the FDA prior to 2021 with molecular weight ≤2000 g/mol were downloaded from e-Drug3D (https://chemoinfo.ipmc.cnrs.fr/MOLDB/index.php). The structures were then optimized using Open Babel in PyRx. Subsequently, proteins structures were downloaded from (https://www.rcsb.org/). Afterward, using AutoDockTools (AD T, 1.5.6), the structures were prepared for the molecular docking study, and nonessential chains, water, and ligand molecules, if present, were eliminated. The final structures were converted into PDBQT format. Docking simulations for all ligands in the prepared library and the selected proteins (NR4A1, DUSP1, and FOS) was performed by AutoDock Vina 1.1.2 in the PyRx software. Visual illustration of the drug-protein interactions were generated with Discovery Studio visualizer 19.1.0.219 (https://discover.3ds.com/discovery-studio-visualizer-download).

## Results

### Exogenous αSN related (EASR) genes have functional links to PD-related genes

**FIGURE 2:**
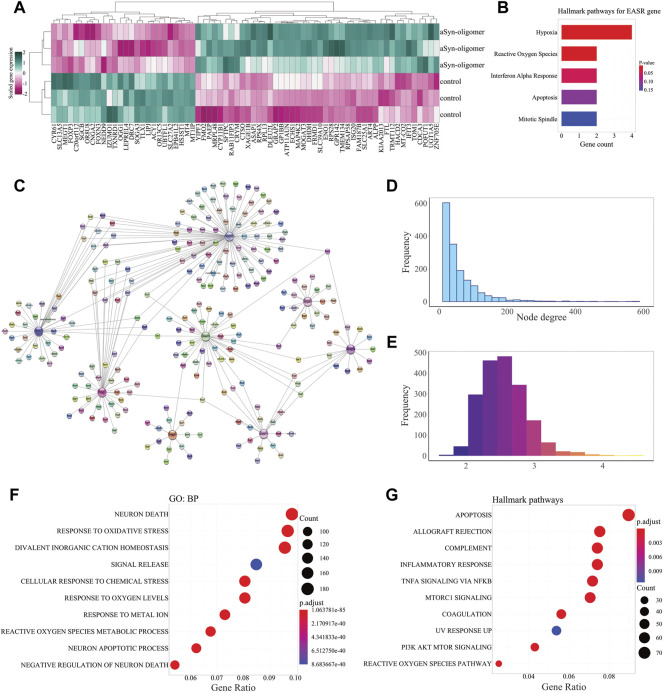
EASR genes and their functional relation to PD-related genes. **(A)** Heatmap of the scaled expression level of differentially expressed genes (*p*-value < 0.05, and fold change > 1.5) in response to exogenous αSN oligomers. Heatmap displays 44 upregulated and 26 down-regulated genes that are considered EASR genes. **(B)** Hallmark pathway enrichment analysis represents EASR genes are significantly enriched only for hypoxia, reactive oxygen species pathway, and interferon-alpha response. **(C)** The PD-EASR subnetwork of 8 overlapped genes (between PD-related and EASR genes) and their connections. The PD-EASR network was constructed and visualized by StringApp in the Cytoscape software, which represents protein-protein interactions between PD-related and EASR genes. **(D)** The Degree distribution shows that most nodes have only small degrees, while a few nodes have vast degrees. This property indicates that the PD-EASR network is a scale-free network. **(E)** Average shortest path length frequency. The PD-EASR network’s average shortest path length-frequency shows that 91.8% of the nodes were communicated together by less than three steps. This feature confirms that the PD-EASR network has functional convergence. **(F)** GO: BP and **(G)** Hallmark pathway enrichment analysis. Neuron death (GO:0070997) and Apoptosis pathway are the most significantly enriched GO: BP and Hallmark pathway.

To identify the genes which were differentially expressed (*p*-value < 0.05, and fold change >1.5) in response to exposure to exogenous αSN aggregate species, we used GSE120569 datasets (Lin et al., 2018). Microarray data analyses led to discovery of 70 DEGs, including 44 upregulated and 26 down-regulated genes (Figure 2A and Supplementary Data S1). We consider these exogenous αSN response genes (EASR genes). Enrichment analysis revealed that EASR genes were primarily associated with the “hypoxia”, “reactive oxygen species” pathway, and “interferon alpha response” (Figure 2B and Supplementary Data S1).

The results obtained from SH-SY5Y cell line as a catecholaminergic line which is able to differentiate to adult neuronal phenotypes including dopaminergic, adrenergic, or cholinergic. Therefore, we first investigated whether the collected EASR genes were specific for dopaminergic, adrenergic, or cholinergic neurons. Our results showed that none of the EASR genes was specific marker for each phenotype of neuron cells (Supplementary Data S2). Accordingly, regardless of the cell model used, the EASR genes could be employed to examine the response to αSN in the different cell types in the brain.

To identify functional links between PD and EASR genes, we obtained PD-related protein-coding genes from the DisGeNET database. We then constructed a PD-EASR network, employing 70 EASR and 1917 PD-related genes (Supplementary Data S3, our data updated on 08/22/2021). The final PD-EASR network contained 1929 nodes (PD-related and EASR genes) and 49,654 edges (Supplementary Figure S1 and Supplementary Data S4). This network represented the protein-protein interactions between 66 EASR genes and 1871 PD-related, that 8 of them were common between the EASR and PD-related genes, including *TXNRD1*, *ASAP1*, *ISG20*, *OGG1*, *FTL*, *NEDD9*, *PAEP*, and *GRAP2* (Figure 2C). In addition, the PD-EASR network contained 66 EASR genes, of which 45 EASR genes did not show any protein interaction with each other. Therefore, EASR genes may play intermediate roles in the PD-EASR network, which links to PD-related genes rather than directly interacting with each other.

To further study the topological characteristics of the PD-EASR network, and the functional links between PD-related and EASR genes were demonstrated. We carried out a network analysis in which we measured topological parameters, such as degree and the average shortest path length. In the PD-EASR network, the degree distribution revealed that many of nodes have a small number of degrees (655 nodes with degree of 1–19), and only a few nodes have high number of degrees (59 nodes with degree >200), suggesting that the PD-EASR network is a scale-free and very robust network (Figure 2D). Additionally, the frequency of the average shortest path length revealed functional convergence among the PD-EASR network, so that almost 91.8% of the nodes were linked by less than three steps (Figure 2E).

To uncover the functional activity of this biological network, we performed enrichment analysis for the core PD-EASR network. We observed that neuron death (GO:0070997) and response to oxidative stress (GO:0006979) were the most significant GO: BP of the PD-EASR network (Figure 2F and Supplementary Data S4). Oxidative stress and cell death are two common features of PD (Dionisio et al., 2021, Amaral and Rodrigues, 2021). Moreover, pathway enrichment analysis highlighted other pathways, including “allograft rejection”, “coagulation”, “complement”, “inflammatory response”, “TNF-α signaling *via* NF-κB”, “PI3K AKT mTOR, mTORC1 signaling”, and “UV response UP” (Figure 2G and Supplementary Data S4).

We conclude that the EASR genes are linked to neuronal death through neurotoxicity pathways, such as oxidative stress and inflammatory-related signaling pathways.

### Expression pattern of the EASR genes associate with different regions of the brain

To find more clues about the reasons for different responses to toxic αSN at various parts of the brain, we explored 1) the expression pattern of EASR genes in the brain regions, and 2) identified co-expression modules and networks that are involved in each region, and also 3) determined co-expression network-related pathways which could contribute to αSN vulnerability. We also studied the putative roles of αSN-related genes and brain region co-expression networks in PD.

**FIGURE 3:**
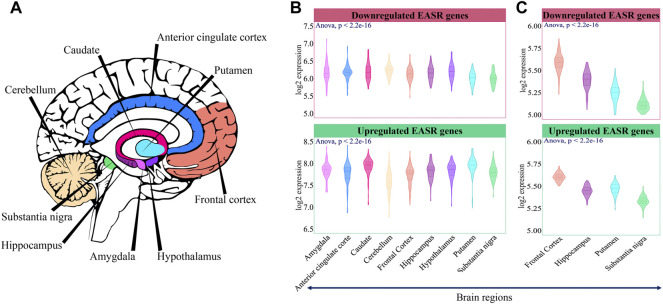
Expression pattern of EASR genes in different brain regions. **(A)** 9 brain regions that were selected for this study. **(B)** Mean expression of downregulated and upregulated EASR genes in each brain region of the GTEx non-neurological healthy samples (illustrated by colors). The expression level of both downregulated and upregulated EASR genes were significantly different across GTEx brain regions (ANOVA, *p*-value < 0.05). **(C)** Validation across healthy samples in UK Brain Expression Consortium (UKBEC). Downregulated and upregulated EASR gene expression levels were significantly different across UKBEC brain regions (ANOVA, *p*-value < 0.05). Putamen was represented the higher expression levels of upregulated EASR genes than substencia nigra in both datasets.

Firstly, we examined the expression level of these genes in different brain regions. GTEx data (https://gtexportal.org/home/) was utilized to examine the expression pattern of the EASR genes in 9 brain regions, including the amygdala, anterior cingulate cortex, caudate, cerebellum, frontal cortex, hippocampus, hypothalamus, putamen, and substantia nigra (Figure 3A). The Sixty-nine upregulated and downregulated EASR genes (Figure 2A and Supplementary Data S1) were mapped on the GTEx expression matrix (all genes except OR8U8), and we observed significant differences in the mean expression of both groups of genes (Figures 3A,B, ANOVA, *p*-value < 0.05).

We observed that some parts, such as substantia nigra, frontal cortex, and cerebellum had the lowest mean expression level in the group of upregulated genes (Figure 3B). As mentioned, degeneration of dopaminergic neurons in the substantia nigra is a primary symptom of PD. Besides, during PD, cerebellum loses its neurological function (Azizi, 2021). On the other hand, caudate and putamen had the highest expression level of upregulated EASR genes (Figure 3B). Note that oxidative damage and mitochondrial dysfunction are reported to be lower in caudate and putamen than in substantia nigra (Venkateshappa et al., 2012). Different expression levels of upregulated EASR genes in the substantia nigra and putamen were also observed in the UKBEC dataset (Figure 3C). Therefore, it seems that the levels of upregulated EASR genes in these brain regions may play important roles in the sensitivity to αSN aggregates.

Moreover, based on GTEx data, there were a highly heterogeneous expression pattern of EASR genes in different regions of the brain (Supplementary Figure S2), suggesting EASR genes may not be functionally homogeneous. In the next step, to examine whether EASR genes could be functionally associated with each other, we measured Spearman’s correlation in each region of the brain. Although most EASR genes showed significant (*p*-value < 0.05) correlations with each other, their correlation coefficients were weak to moderate (correlation coefficient values (r) between |0.1| and |0.4|) (Supplementary Figure S3), suggesting that EASR genes had separate functional modules. Consequently, it seems that the expression level of individual EASR genes is not sufficiently indicative to identify the causes of PD vulnerability in different regions.

### EASR co-expression modules are associated with PD and enriched for protein processing and COVID-19 related terms

**FIGURE 4:**
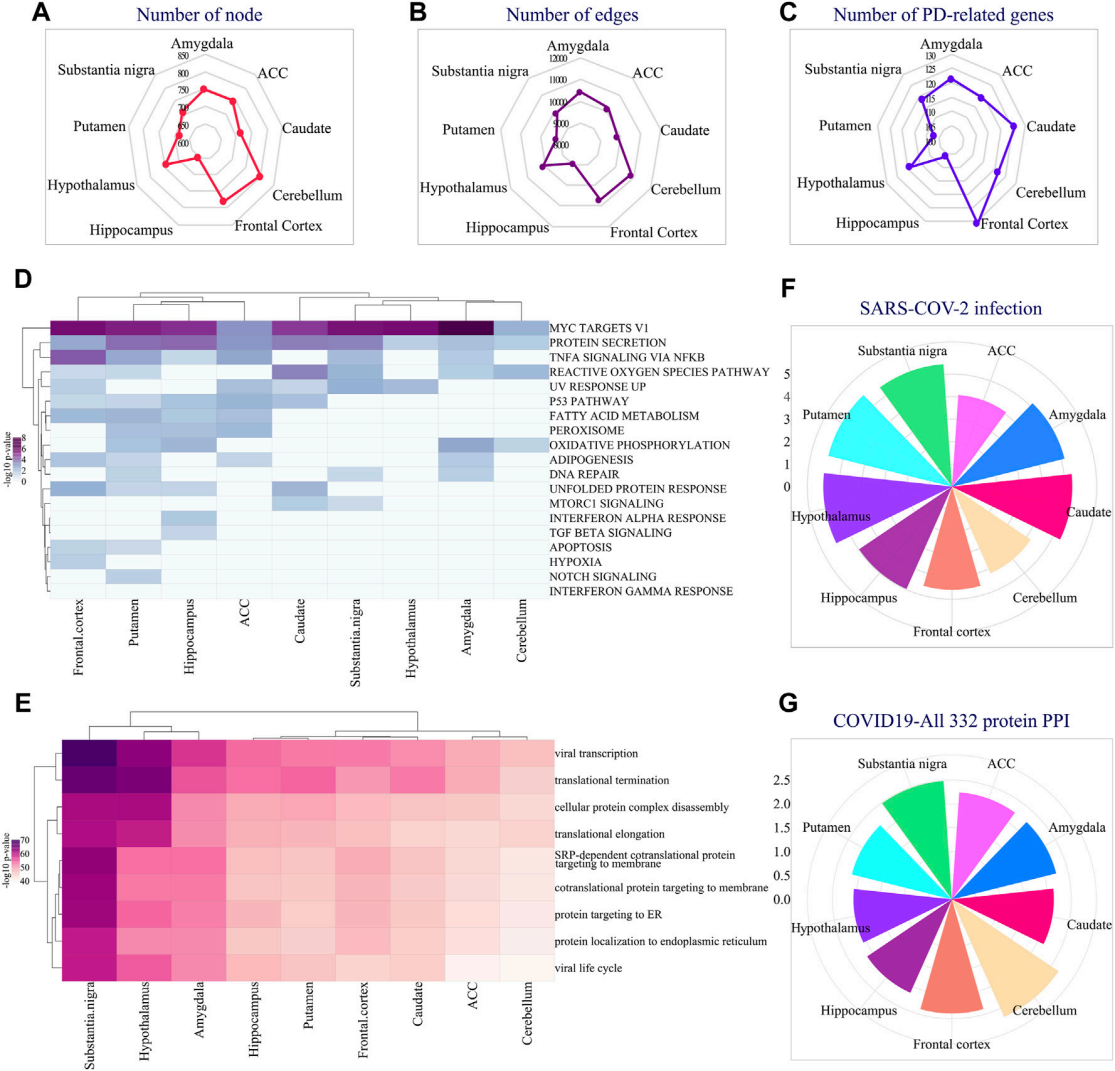
Analysis of region-specific EASR genes co-expression networks. Radar plot demonstrating the number of **(A)** co-expression network’s nodes and **(B)** Edges in different brain regions. **(C)** Radar plot showing the number of PD-related genes present in each region’s specific co-expression network. **(D)** Hallmark pathway enrichment analysis of co-expression network, across all 9 brain regions, illustrated by heatmap of -log10 adj *p*-value. **(E)** GO: BP enrichment analysis of co-expression network, across all 9 brain regions, illustrated by heatmap of -log10 adj *p*-value. Also, the “COVID-19 Related Gene Sets 2021″ enrichment analysis represented all EASR co-expression networks enriched for some important COVID-19 related terms, such as **(F)** Genes whose expression is altered by SARS = COV-2 infection, and **(G)** “COVID19-All 332 protein host PPI” gene sets. F-G circular bar plots demonstrating -log10 adj *p*-value of each term.

In addition to the region-specific expression of EASR genes, we extracted and analyzed co-expressed networks for all 9 regions of the brain and examined their functional annotation. We reconstructed networks by top 25 co-expression genes using the Inetmodels (considering positive and negative correlations) for each region. The constructed networks had 654–808 nodes and 9,007–10889 edges, respectively (Figures 4A,B). We also examined whether each region-specific co-expression network has a common sub-network with the PD-EASR network. Our study identified on average 119 common nodes and 4.7% similarity between the PD-EASR and each co-expression network among 9 regions (Figure 4C and Supplementary Figure S4). Therefore, in each brain region, the expression of EASR genes significantly correlated to some of the PD-related genes (Supplementary Figure S5). Consequently, exogenous aggregated αSN leads to changes in the genes set that are associated with PD through both the PPI network and the EASR co-expression network in every brain region.

Additionally, we performed enrichment analysis for all EASR co-expression networks. We found that genes that correlated to EASR genes significantly enriched for “MYC targets V1”, “protein secretion”, “TNF-α signaling via NF-κB”, and “reactive oxygen species pathway” (Figure 4D and Supplementary Data S5).

Moreover, all the EASR co-expression networks were associated with protein processing related GO-terms, such as “translational termination” (GO:0006415), “cellular protein complex disassembly”, “translational elongation”, “co-translational protein targeting to the membrane” (GO:0006613), “protein targeting to ER” (GO:0045047), and “establishment of protein localization into the endoplasmic reticulum” (Figure 4E and Supplementary Data S5). Interestingly, we also observed that co-expression networks were significantly enriched for viral-related processing, such as viral transcription (GO:0019083) and viral life cycle (Figure 4E and Supplementary Data S5).

Accordingly, we were interested in examining possible links between the EASR co-expression network and COVID-19. Several studies have confirmed a link between COVID-19 and PD (Antonini et al., 2020; Cilia et al., 2020; Leta et al., 2021). Furthermore, it has been suggested that the dopamine synthesis pathway is involved in the pathophysiology of COVID-19 (Antonini et al., 2020). However, the molecular mechanisms of association between PD and COVID-19 have not been fully understood. Therefore, we investigated whether the αSN could be associated with the pathophysiology of COVID-19. To this end, we performed a “COVID-19 Related Gene Sets 2021” enrichment analysis and observed that all EASR co-expression networks encompass genes whose expression levels are altered by SARS-CoV-2 infection (Figure 4F and Supplementary Data S5).

In addition, affinity-purification MS studies have identified 332 high-confidence protein-protein interactions between human proteins and SARS-CoV-2 (Gordon et al., 2020). Targeting these proteins might have therapeutic potential against COVID-19 infection (Gordon et al., 2020). We observed all EASR co-expression also significantly enriched for “COVID19-All 332 protein host PPI” gene sets that reveal a link between PD and COVID-19 through αSN aggregates (Figure 4G and Supplementary Data S5).

### αSN-related co-expression network revealed the role of TNF-α signaling in PD

**FIGURE 5:**
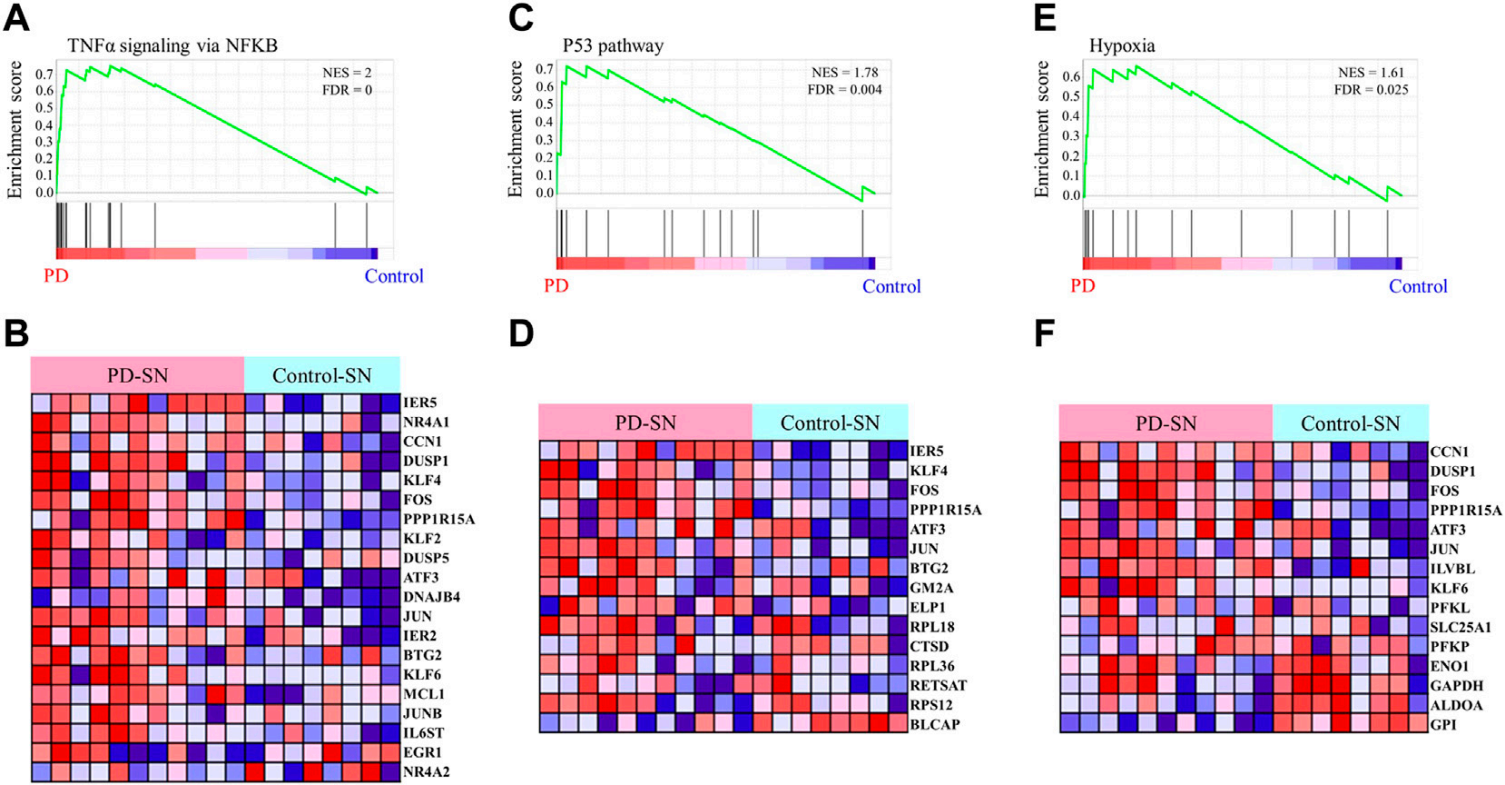
Gene Set Enrichment Analysis of the EASR co-expression network. The analysis demonstrates that TNF-α signaling via NF-κB **(A,B)**, P53 pathway **(C,D)**, and hypoxia **(E,F)** are significantly enriched in substantia nigra of PD phenotype. FDR <0.05 was considered as a statistically significant level.

To identify the pathological function of the EASR co-expression network in substantia nigra, we used the GSE114517 dataset and performed GSEA between PD and healthy control (HC) conditions. We observed that the EASR co-expression network was significantly (FDR <0.05) enriched and up-regulated in 3 hallmark pathways (Figure 5 and Supplementary Data S6), including “TNF-α signaling via NF-κB” (NES = 2.00, FDR = 0), “P53 pathway” (NES = 1.78, FDR = 0.004), and “hypoxia pathway” (NES = 1.61, FDR = 0.025). We also performed GSEA using all coding genes in substantia nigra to elucidate whether the three observed pathways were the main pathways that dysregulated during PD. We identified 16 pathways that were significantly upregulated in PD, including “TNF-α signaling *via* NF-κB” (NES = 2.2974796), “IL6 JAK STAT3 signaling” (NES = 2.1603885), “allograft rejection” (NES = 1.9858677), “inflammatory response” (NES = 1.8908404), “epithelial-mesenchymal transition” (NES = 1.850037), “angiogenesis” (NES = 1.8474189), “TGFβ signaling” (NES = 1.7642163), “interferon-gamma response” (NES = 1.6653343), “hypoxia” (NES = 1.626201), “complement” (NES = 1.6150334), “P53 pathway” (NES = 1.5878932), “apoptosis” (NES = 1.5682892), “KRAS signaling UP” (NES = 1.56), “IL2 STAT5 signaling” (NES = 1.56), “coagulation” (NES = 1.53), and “interferon alpha response” (NES = 1.4) (Supplementary Figure S6 and Supplementary Data S7). Consequently, a region-specific EASR co-expression network is associated with the upregulation of main pathways in the pathogenesis of PD.

We also applied the same analysis for the EASR co-expression network in the amygdala and, interestingly, found no pathway to be significantly enriched in PD phenotype (Supplementary Data S6). Therefore, these results suggest that the vulnerability to the toxic forms of αSN in the substantia nigra is because of region-specific gene expression patterns and an EASR-related co-expression network.

### Drug discovery

**FIGURE 6:**
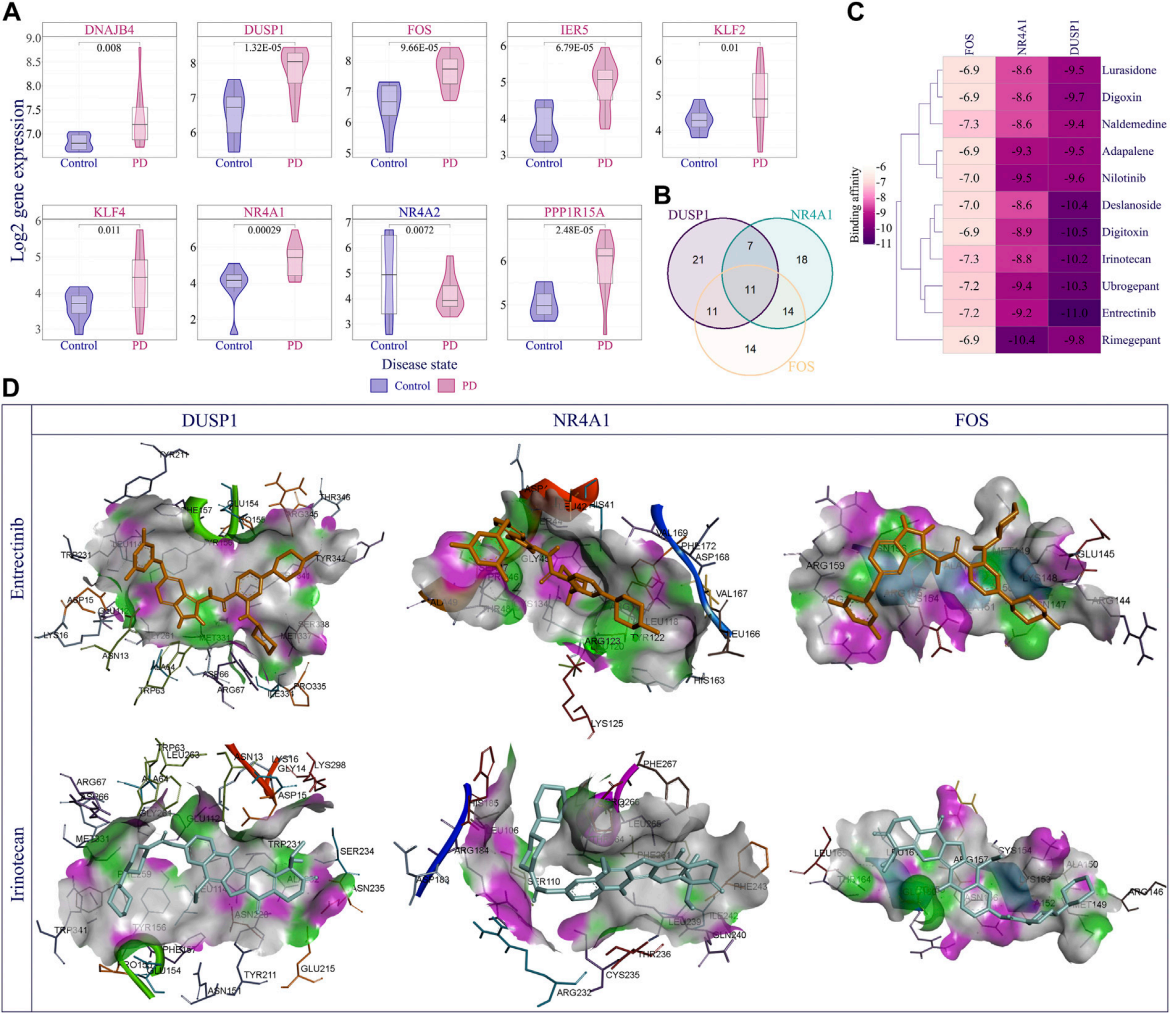
Discovery of best inhibitors for TNF-α signaling via NF-κB pathway. **(A)** The violin plot demonstrates the distribution of log2 expression of nine dysregulated genes (|fold change | > 1.5 and *p*-value < 0.05) in the TNF-α signaling via NF-κB pathway in the PD compared to the control samples. **(B)** Venn diagram represents the overlapping top 50 inhibitors between NR4A1, DUSP1, and FOS. Eleven overlapping drugs were obtained for further analysis, including adapalene, Deslanoside, Digitoxin, Digoxin, Entrectinib, Irinotecan, Lurasidone, Naldemedine, Nilotinib, Rimegepant, and Ubrogepant. **(C)** Heatmap showing Affinity binding energy between common eleven inhibitions and NR4A1, DUSP1, and FOS proteins. **(D)** The visual illustration showed the binding energy of Entrectinib and Irinotecan with studied proteins. The docked pose of drugs and each protein showed the key hydrogen-bonds area using docking results generated with Discovery Studio visualizer 19.1.0.219 as a free resource.

Finally, we performed DEG analysis and observed that 66 nodes of the EASR co-expression network in substantia nigra were significantly dysregulated. Across the EASR co-expression network, nine genes were dysregulated in the “TNF-α signaling via NF-κB” pathway, of which eight genes were upregulated, including *DNAJB4*, *KLF2*, *KLF4*, *PPP1R15A*, *FOS*, *DUSP1*, *IER5*, and *NR4A1*, and only *NR4A2* were downregulated (Figure 6A). Since the “TNF-α signaling *via* NF-κB” pathway was the main enhanced pathway during PD and was the significantly enriched pathway for eight brain regions, it seems that inhibition of this pathway can be an appropriate approach to counteract the neurotoxic effect of αSN and also reduce the severe effects of COVID-19 on PD patients. However, our study revealed that except for FOS, none of the upregulated genes were targeted by FDA-approved drugs. Of the two FDA-approved drugs against FOS (nadroparin and pseudoephedrine), only Pseudoephedrine can cross the blood-brain barrier (BBB). Therefore, we conducted virtual screening on FDA-approved drugs to identify new inhibitors for NR4A1 (PDB: 3V3E), DUSP1 (PDB: 6D66), and FOS (PDB: 1S9K) using their available structures. We performed docking simulations and then ranked the drugs according to binding affinity, focusing on the top 50 ranked drugs. Next, the common drugs considered as “TNF-α signaling *via* NF-κB” pathway inhibitors (Figure 6B and Supplementary Data S8). Our results represented 11 overlap drugs (Figure 6B) with the highest binding affinity between NR4A1, DUSP1, and FOS, including adapalene (DB00210), deslanoside (DB01078), digitoxin (DB01396), digoxin (DB00390), entrectinib (DB11986), irinotecan (DB00762), lurasidone (DB08815), naldemedine (DB11691), nilotinib (DB04868), rimegepant (DB12457), and ubrogepant (DB15328) (Figures 6B,C). Based on DrugBank information (https://go.drugbank.com/), 5 drugs (adapalene, entrectinib, irinotecan, lurasidone, and nilotinib) cross BBB and during PD could find use as inhibitors of the “TNF-α signaling *via* NF-κB” pathway. Docking simulations indicated entrectinib and irinotecan had the lowest negative energy for NR4A1, DUSP1, and FOS (Figures 6C,D).

## Discussion

PD is the second most common age-related neurodegenerative disorder, characterized by a broad spectrum of motor and non-motor symptoms. So far, there are no effective preventive or curative therapies for PD (Iarkov et al., 2020), and current treatments only counteract dopamine loss. Thus, approved therapies target the final phenotypes and signaling cascades that cause neuronal death instead of the primary elements.

αSN is one of the principal factors associated with the onset and progression of PD (Davidi et al., 2020). However, the underlying molecular mechanisms of αSN toxicity and the spatial distribution of neuronal loss in the brain are not well understood. Here we used an microarray study (GSE120569) to identify EASR genes. We reasoned that these genes, which are affected by αSN aggregation, might provide insights into molecular mechanisms of αSN toxicity and lead to effective treatments. Therefore, we applied systems biology approaches to characterize EASR genes functions in different brain regions and their association to PD.

In the first step, we constructed a PD-EASR network by mapping PD-related and EASR genes on the human PPI network. Subsequently, we examined the topological features of the PD-EASR network. This revealed that the PD-EASR network is a scale-free network, which is a common characteristic of biological networks (Uversky and Giuliani, 2021). Also, to fully understand the functional links between PD-related genes and EASR genes, we performed enrichment analysis for the core PD-EASR network, representing its function in neuron death (GO:0070997). The pathway enrichment analysis suggested that the association between “apoptosis”, “inflammatory response”, and “reactive oxygen species” pathways through the core PD-EASR network leads to loss of neurons in PD.

By identifying the functional connection between EASR genes and PD, we next examined the expression of these genes in different brain regions to determine whether EASR genes have region-specific expression patterns. We observed that the mean expression of upregulated and downregulated EASR genes were significantly different across all 9 regions of a healthy brain. Specifically, cerebellum, frontal cortex, anterior cingulate cortex, and substantia nigra, showed the lowest mean expression level of upregulated-EASR genes. Although cerebellum function in PD has not been fully clarified, it has been demonstrated that morphological and functional modifications of the cerebellum are associated with αSN aggregation in patients with synucleinopathies (Seidel et al., 2017), and its neurological function would be lost during disease progression (Azizi, 2021). Furthermore, the frontal cortex is dysfunctional in PD, leading to cognitive impairment in these patients, such as visuospatial dysfunction or slowed thinking (Parker et al., 2013). Likewise, there is disruption of anterior cingulate cortex activity in PD patients, which could be connected to language or executive dysfunctions in PD (Vogt, 2019). More recently, cortical dysfunction has been shown to be involved in the PD-induced chronic pain model in mice (Zhou et al., 2021). Therefore, it seems that the lower expression level of upregulated-EASR genes (under healthy conditions) could contribute to higher vulnerability to exogenous aggregated αSN.

On the other hand, caudate and putamen (which compose the dorsal striatum) had the highest expression level of upregulated-EASR genes. The striatum is connected to the substantia nigra through the nigrostriatal pathway, one of the four major dopamine pathways (Iyer et al., 2021). However, unlike the substantia nigra, it has been reported that oxidative damage and mitochondrial dysfunction in the striatum (caudate and putamen) were lower in the PD brains (Venkateshappa et al., 2012). To confirm these results, we used UKBEC expression data, which reported on 4 regions of our studied brain regions. Thus, our findings of UKBEC only confirmed the higher expression level of upregulated-EASR genes in putamen compared to substantia nigra. Consequently, it seems that with the increased expression level of upregulated-EASR genes, brain regions are more resistant to the exogenous αSN-induced changes, specifically among substantia nigra and putamen, which are connected to each other. However, collecting and studying more samples from healthy and PD brains regions in the future could provide a more accurate view of the expression pattern of EASR genes and their functions in different brain regions.

We also extracted EASR genes co-expression modules, analyzed co-expressed networks for the healthy brain regions, and then determined which pathways of substantia nigra co-expression network are changed during PD. The “TNF-α signaling via NF-κB” pathway turned out to be the most significant upregulated pathway (FDR = 0 and NES = 2.3) in the substantia nigra of PD patients. This result is supported by other *in vivo* and *in vitro* studies that show NF-κB to be dysregulated and activated in PD (Baiguera et al., 2012; Liu et al., 2014; Parrella et al., 2019; Bellucci et al., 2020; Wang et al., 2020). Furthermore, it has already been shown that active phosphorylated NF-κB (or RelA) is present in the nucleus of neurons and glial cells of the substantia nigra in PD patients (Ghosh et al., 2007; Garcia-Esparcia et al., 2014). Some studies suggested that NF-κB is affected in αSN -related neuronal loss (Togo et al., 2001; Bellucci et al., 2020). However, the molecular connection between αSN aggregation and TNF-α signaling via the NF-κB pathway has not been fully elucidated.

Pathway enrichment studies on EASR genes showed no association between EASR genes and the “TNF-α signaling *via* NF-κB” pathway. However, this association was seen in both the PD-EASR network and region-specific EASR co-expression network in healthy substantia nigra, amygdala, cortex, and putamen. Although the genes that altered in response to exogenous αSN were not directly involved in the “TNF-α signaling *via* NF-κB” pathway, they could be connected and could affect this pathway through a protein/region-specific co-expression network. Given that the EASR co-expression network in the amygdala has no significant alteration in this signaling pathway, it seems that the region-specific gene expression pattern in substantia nigra leads to higher vulnerability to exogenous αSN in PD.

Identifying and understanding molecular mechanisms behind the toxicity of αSN aggregation could pave the way towards more efficient treatments for PD patients. Hence, drugs that inhibit the TNF-α signaling via the NF-κB pathway, as a key pathway involved in αSN toxicity, could be an effective therapeutic approach for PD. Furthermore, our results represented EASR genes co-expression networks as a molecular mechanism to link PD and COVID-19. It has already been reported that PD patients with COVID-19 present aggravated parkinsonian symptoms, and higher mortality has been reported in patients with advanced PD (Antonini et al., 2020; Cilia et al., 2020; Sulzer et al., 2020; Zhang et al., 2020; Leta et al., 2021). Nevertheless, there is still controversy over this, and the effect of COVID-19 on increasing mortality in PD patients is still debated (Fearon and Fasano, 2021). Accordingly, considering the COVID-19 pandemic, modifying a substantial nigra-related co-expression network by inhibiting TNF-α signaling via the NF-κB pathway, as the most significant upregulated pathway during PD, could also reduce COVID-19 symptoms in PD patients. Our virtual screening identified several FDA-approved drugs that could inhibit three upregulated proteins in this pathway, including NR4A1, DUSP1, and FOS.

Targeting the αSN neurotoxicity pathway runs counter to most existing treatments that try to restore the dopaminergic system (Iarkov et al., 2020). Therefore, our treatment strategy could inhibit the primary signaling cascades which lead to PD symptoms. Moreover, multiple targets screening, such as targeting multiple proteins of a pathway or multiple pathways of disease, is a novel concept for drug repurposing, which, like drug combination screening, could increase therapeutic efficacy (Cheng, Kovács and Barabási, 2019; Liang et al., 2021). Accordingly, we performed screening on FDA-approved small molecules and identified 11 common drugs with significant binding affinity for NR4A1, DUSP1, and FOS proteins. Among these entrectinib and irinotecan had the lowest binding energy for NR4A1, DUSP1, and FOS and can cross the BBB, making them potential candidates for PD treatment.

Another point is that this study was based on the SH-SY5Y cells line only. However, our examinations have shown that the EASR genes are not specific markers for dopaminergic, adrenergic or cholinergic neuron phenotypes. Therefore, regardless of the cell model, we could use the EASR genes for subsequent analyses. We reasoned that these genes could provide insights into molecular mechanisms of αSN toxicity and lead to discover new effective treatments. Moreover, in the next step of our study, we used the expression data of healthy brain tissue with various types of cells. Therefore, the effect of cell-specific pathways that are related to each glia or neuron cells has not been evaluated and needs more studies in the future.

In conclusion, we determined functional cross-links between EASR and PD-related genes in PPI and region-specific co-expression networks. Our finding identified genes and pathways that could be involved in toxicity and different vulnerability to exogenous αSN. Furthermore, we highlighted exogenous αSN effects in different brain regions by characterizing interactions between EASR genes and pathways in healthy brain regions. Also, these results suggest that an increase in the “TNF-α signaling *via* NF-κB” pathway during PD could be one of the effects of exogenous αSN through the EASR genes co-expression network. In total, our observations provide a better insight into the pathology of exogenous αSN and may point the way towards future new treatments.

## Data Availability

The datasets presented in this study can be found in online repositories. The names of the repository/repositories and accession number(s) can be found in the article/Supplementary Material.

## Abbreviations

PD, parkinson’s disease; αSN, α-synuclein; EASR genes, exogenous αSN response genes; LBs, lewy bodies; LNs, lewy neurites; GSEA, gene set enrichment analysis; PPI network, protein-protein interaction network; DEGs, differentially expressed genes; GTEx, genotype-tissue expression; UKBEC, UK brain expression consortium; GO, gene ontology; MF, molecular function; BP, biological process; CC, cellular component; MSigDB, broad institute’s molecular signatures database; FDR, false discovery rate; NES, normalized enrichment score.
