# Supplementary material for: Characterization of exogenous αSN response genes and their relation to Parkinson’s disease using network analyses
Source: Front Pharmacol. 2022 Sep 30;13:966760. doi: 10.3389/fphar.2022.966760 (PMC9563388; doi:10.3389/fphar.2022.966760)
Supplement: Supplementary file 4 [file DataSheet1.PDF]

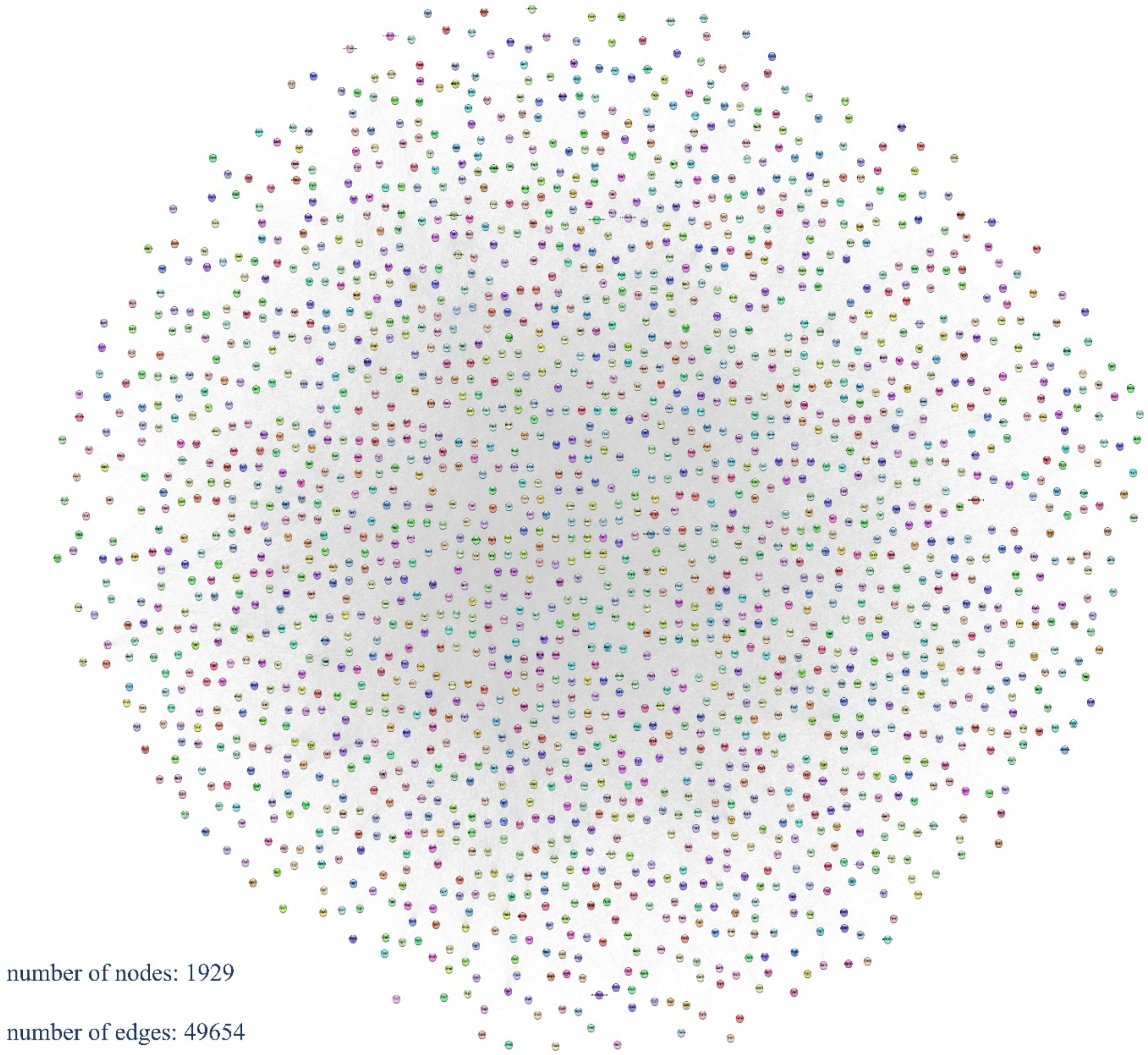

Figure S1. PD-EASR network. The PD-EASR network was constructed and visualized by StringApp in the Cytoscape software. The PD-EASR network contains 1929 nodes and 49654 edges representing protein-protein interactions between PD-related and EASR genes.

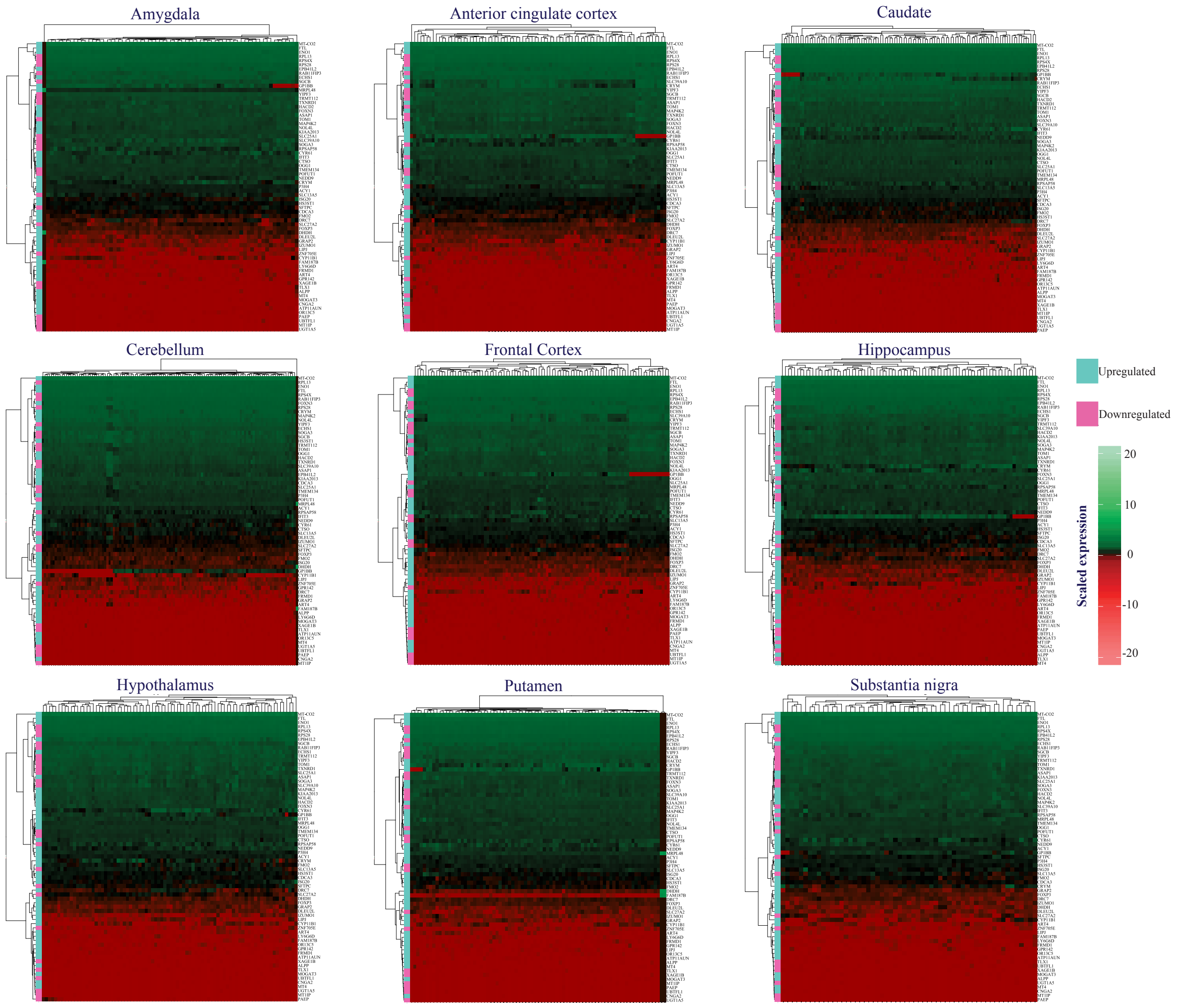

Figure S2. Heatmap of EASR gene expression in 9 brain regions. The ComplexHeatmap R package was performed to illustrate heatmaps. The intensity of the color displays the expression level in each brain region. The color bar plot represented upregulated-EASR genes (pink) and downregulated-EASR genes (blue).

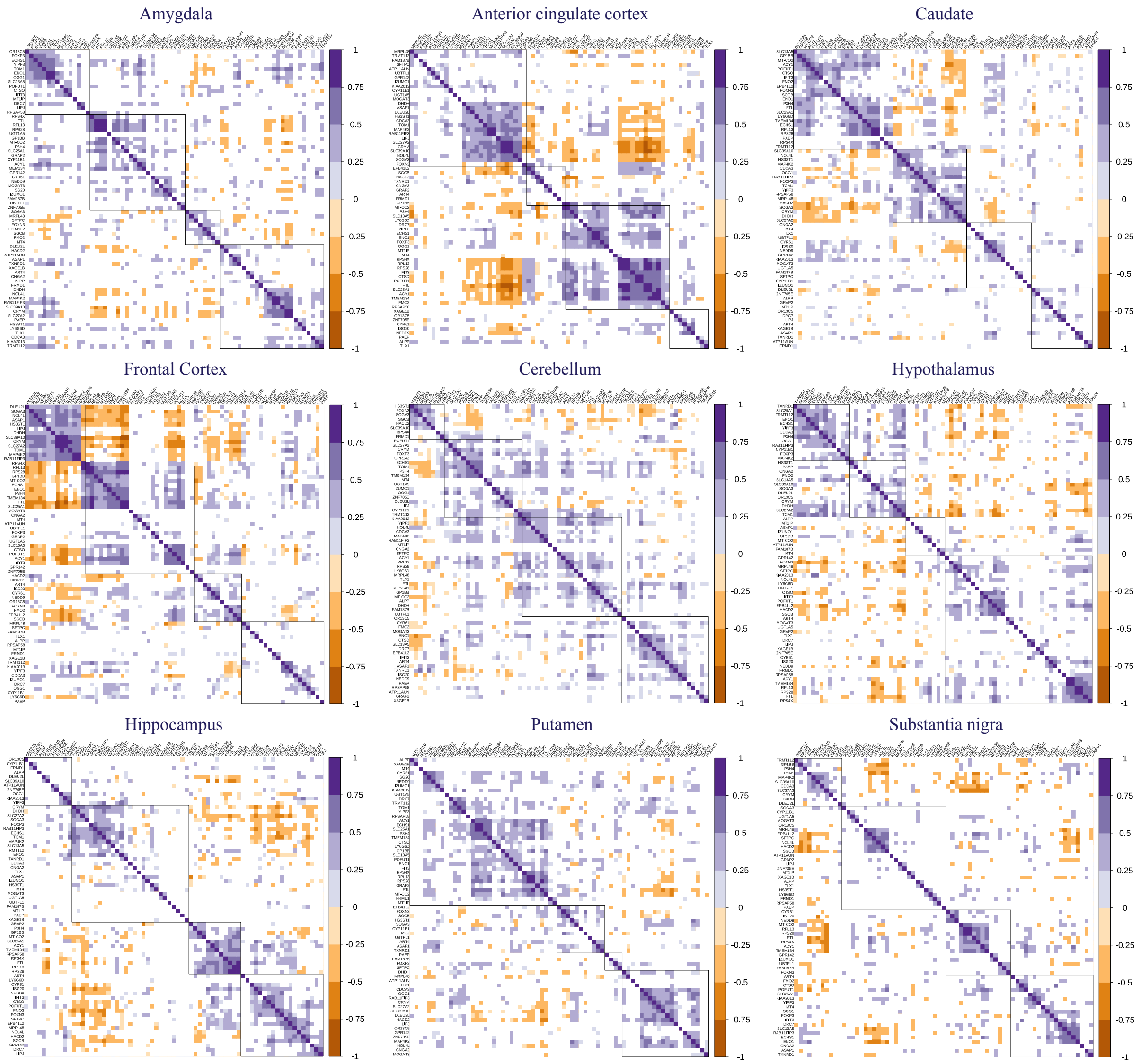

Figure S3. Correlation analyses of EASR genes in 9 brain regions. The Corrplot R package was performed to display spearman's correlation results. The intensity of the color indicates the correlation coefficient in each brain region.

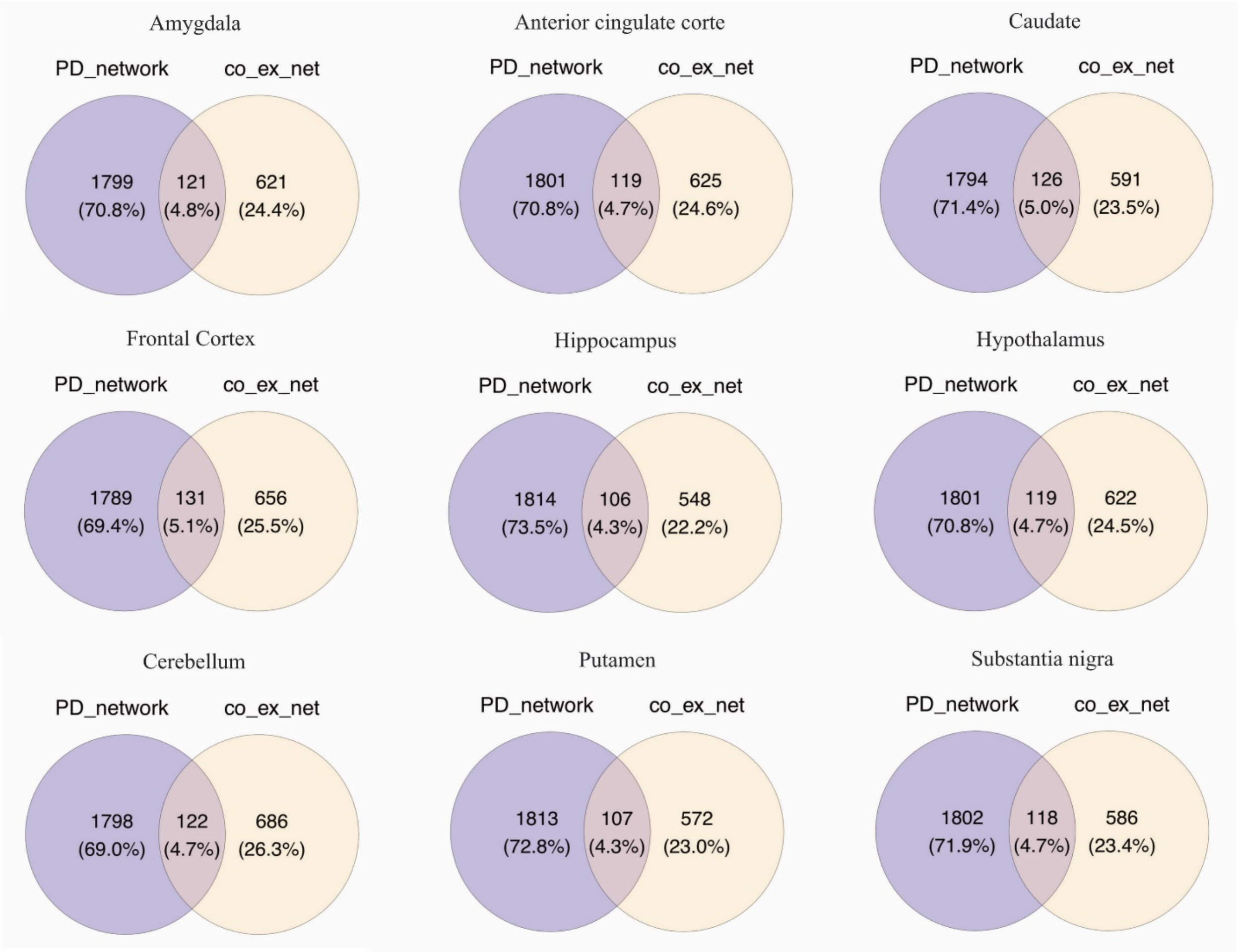

Figure S4. Venn diagram demonstrating overlap between PD-related genes by EASR co-expression genes across 9 brain regions. The VennDiagram R package was used to illustrate Venn diagrams.

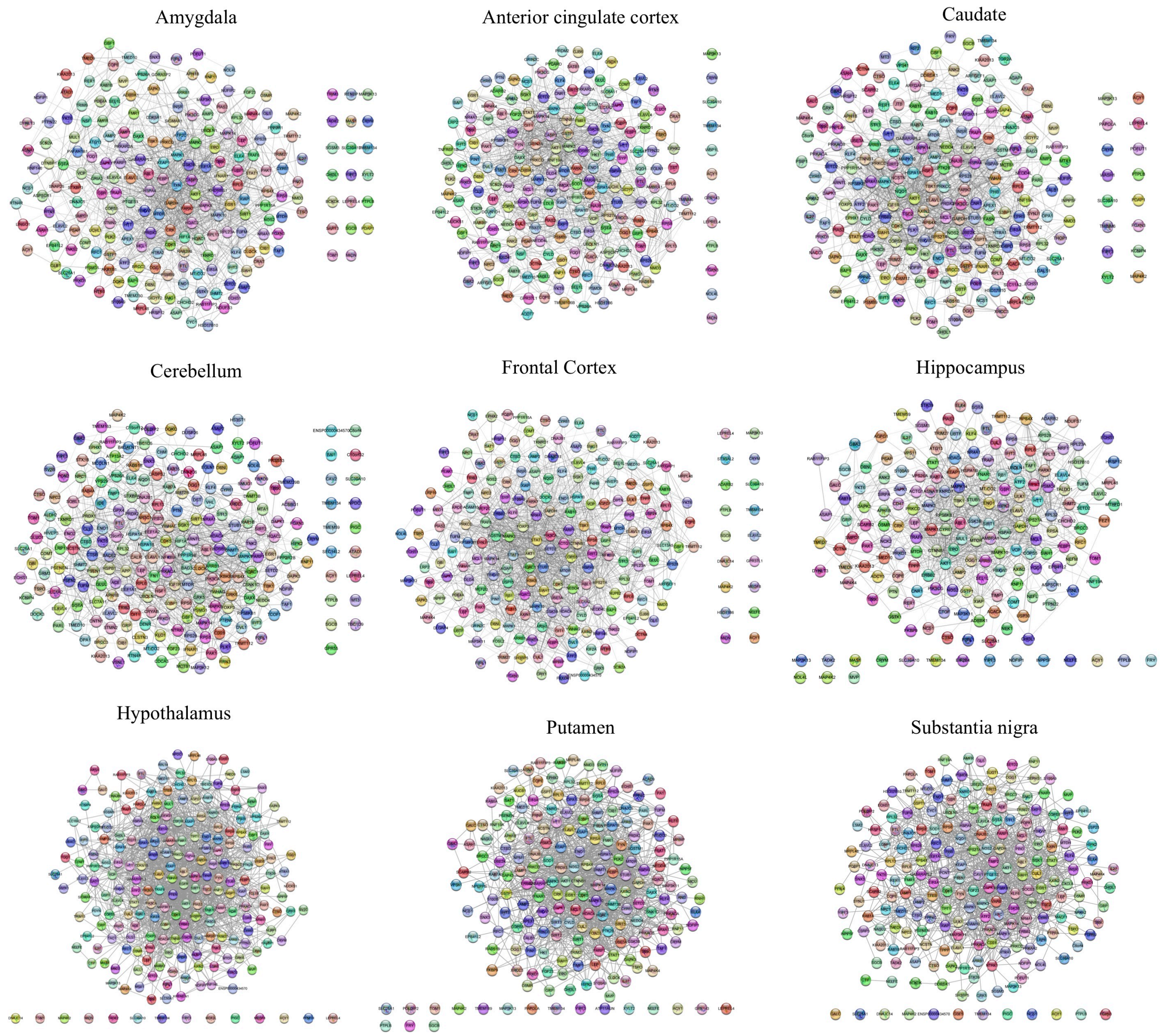

Figure S5. Intersection networks between PD-EASR network and EASR co-expression networks in each brain region. Networks were visualized in the Cytoscape software.

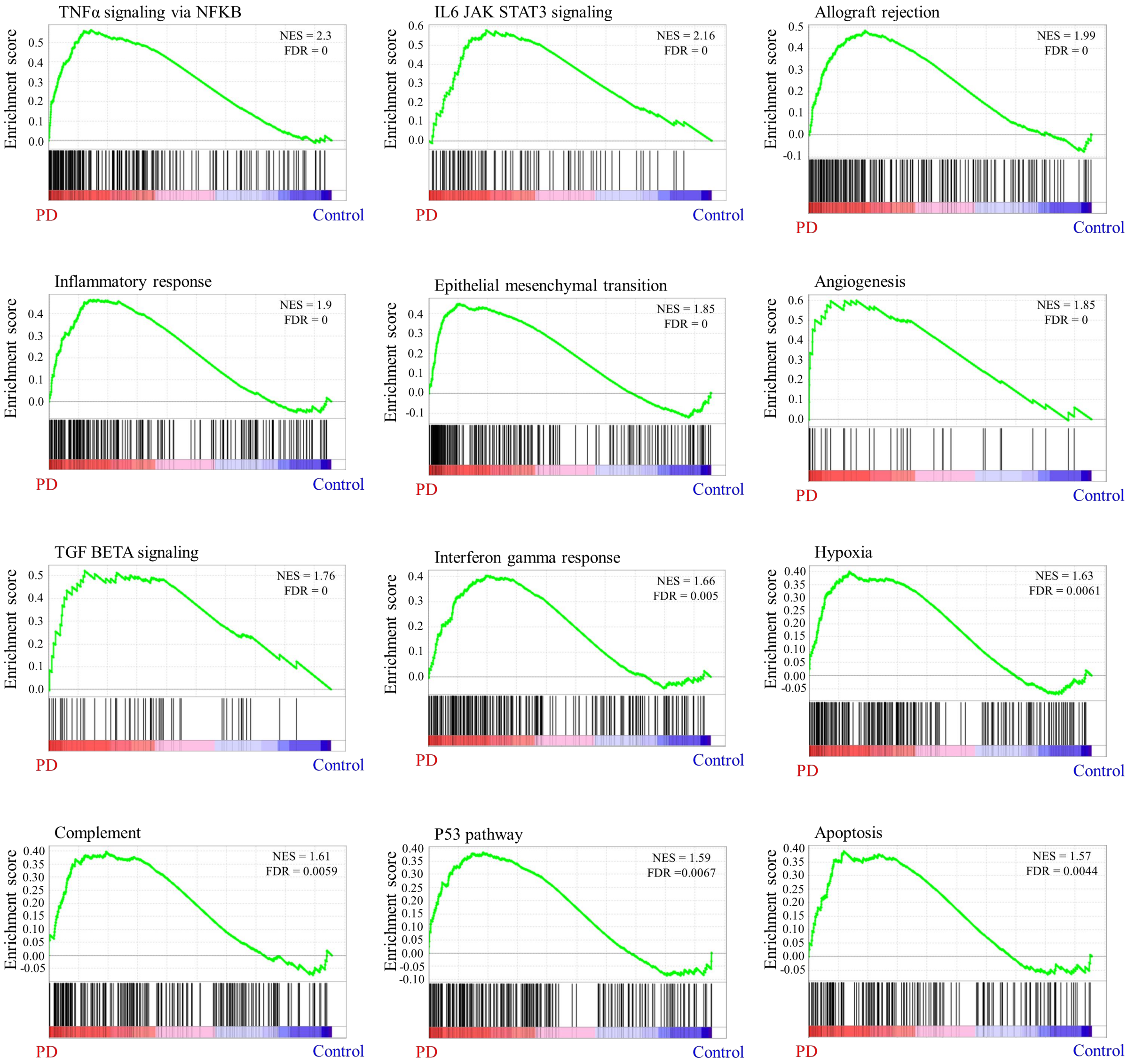

Figure S6. Gene set enrichment analysis between PD and normal samples. The figure represented the top enriched pathways of the hallmark gene set collection from MSigDB.
